# Pyrazine-Based Blue Thermally Activated Delayed Fluorescence Materials: Combine Small Singlet–Triplet Splitting With Large Fluorescence Rate

**DOI:** 10.3389/fchem.2019.00312

**Published:** 2019-05-21

**Authors:** Junyuan Liu, Keren Zhou, Dan Wang, Chao Deng, Ke Duan, Qi Ai, Qisheng Zhang

**Affiliations:** MOE Key Laboratory of Macromolecular Synthesis and Functionalization, Department of Polymer Science and Engineering, Zhejiang University, Hangzhou, China

**Keywords:** thermally activated delayed fluorescence (TADF), pyrazine, blue organic light-emitting diodes (OLED), fluorescence rate constant, singlet-triplet splitting

## Abstract

Metal-free thermally activated delayed fluorescence (TADF) emitters have emerged as promising candidate materials for highly efficient and low-cost organic light-emitting diodes (OLEDs). Here, a novel acceptor 2-cyanopyrazine is selected for the construction of blue TADF molecules via computer-assisted molecular design. Both theoretical prediction and experimental photophysical data indicate a small S_1_-T_1_ energy gap (Δ*E*_ST_) and a relative large fluorescence rate (*k*_F_) in an *o*-phenylene-bridged 2-cyanopyrazine/3,6-di-*tert*-butylcarbazole compound (TCzPZCN). The *k*_F_ value of 3.7 × 10^7^ s^−1^ observed in a TCzPZCN doped film is among the highest in the TADF emitters with a Δ*E*_ST_ smaller than 0.1 eV. Blue TADF emission is observed in a TCzPZCN doped film with a short TADF lifetime of 1.9 μs. The OLEDs using TCzPZCN as emitter exhibit a maximum external quantum efficiency (EQE) of 7.6% with low-efficiency roll-off. A sky-blue device containing a derivative of TCzPZCN achieves an improved EQE maximum of 12.2% by suppressing the non-radiative decay at T_1_.

## Introduction

Owing to the small energy gap (Δ*E*_ST_) between the lowest singlet (S_1_) and triplet (T_1_) excited states, metal-free thermally activated delayed fluorescence (TADF) molecules can upconvert from their T_1_ to S_1_ by absorbing environmental thermal energy and then decay radiatively from the S_1_. Organic light-emitting diodes (OLEDs) employing TADF emitters can convert both singlet and triplet excitons into light with a theoretical yield up to 100% (Wex and Kaafarani, 2017; Yang et al., 2017; Cai and Su, 2018; Cai et al., 2018; Liu Y. et al., 2018), and have emerged as a new representation for highly efficient and low-cost OLEDs (Liu et al., 2014; Li W. et al., 2014; Ai et al., 2018; Bian et al., 2018). A twisted donor–phenylene–acceptor (D-Ph-A) structure has been demonstrated to be an effective strategy for the design of TADF materials (Zhang et al., 2012). A number of efficient blue, green, and red TADF materials with small Δ*E*_ST_ have been developed by using this strategy in recent years (Uoyama et al., 2012; Wang et al., 2014, 2017; Zhang et al., 2014a,b; Chen et al., 2016; Chen X.-L. et al., 2017; Li et al., 2017; Yuan et al., 2017; Wu et al., 2018; Zhang D. et al., 2018).

Except for Δ*E*_ST_, the value of fluorescence rate (*k*_F_) for TADF emitters has attracted more and more attention in recent years, because it is a key for not only the quantum efficiency of the emitter but also the TADF lifetime and the device stability (Zhang et al., 2014a,b; Liu Z. et al., 2018). The D-Ph-A-type TADF emitters with small twisting angles between the neighboring planes can have high *k*_F_ values but suffer from large Δ*E*_ST_, which leads to significant efficiency roll-off in their devices (Zhang et al., 2012; Li et al., 2013; Hirata et al., 2015; Chen X.-K. et al., 2017). Although increasing the twisting angle can reduce Δ*E*_ST_, the *k*_F_ value also decreases due to the reduced overlap of the orbitals involved in the S_1_ transition (Zhang et al., 2014a). Especially, for blue TADF emitters with large band gaps, large twisting angle cannot ensure a small Δ*E*_ST_, because the molecules may have a low-lying triplet state localized at the D or A moieties (Zhang et al., 2014b). Overall, the difficulty of TADF material design is to have small Δ*E*_ST_ and high *k*_F_ at the same time. To enlarge the ratio of *k*_F_ to Δ*E*_ST_, the D-A couple should be carefully selected, and the twisting geometry should be well-designed.

Cyano (Uoyama et al., 2012; Li B. et al., 2014; Lee and Lee, 2015; Taneda et al., 2015; Zhang et al., 2016; Chan et al., 2018; Sommer et al., 2018) and aromatic imines [i.e., triazine (Endo et al., 2011; Lee et al., 2012; Tanaka et al., 2012; Hirata et al., 2015; Kaji et al., 2015; Cha et al., 2016; Lin et al., 2016; Zhu et al., 2016; Chen X.-K. et al., 2017; Cui et al., 2017; Shao et al., 2017; Liu Z. et al., 2018; Oh et al., 2018; Zhang Q. et al., 2018; Wang Q. et al., 2019; Zhang et al., 2019) pyrimidine (Gómez-Bombarelli et al., 2016; Komatsu et al., 2016; Pan et al., 2016; Wu et al., 2016; Ganesan et al., 2017; Nakao et al., 2017; Park et al., 2017; Xiang et al., 2017; Zhang Q. et al., 2018; Zhang et al., 2019), and pyridine (Tang et al., 2015; Pan et al., 2016; Rajamalli et al., 2017; Sasabe et al., 2017; Chen et al., 2018)] are the most used groups for the construction of acceptor moieties in TADF molecules. The electron-withdrawing capability of an aromatic imine increases with an increase in the number of nitrogen atoms in the ring. 2,4,6-Triphenyl-1,3,5-triazine (TRZ) is a promising acceptor for blue and green TADF materials because of the high T_1_ energy level and the relatively strong electron-withdrawing capability (Tanaka et al., 2012; Hirata et al., 2015; Kaji et al., 2015; Cha et al., 2016; Lin et al., 2016; Chen X.-K. et al., 2017; Cui et al., 2017; Shao et al., 2017; Liu Z. et al., 2018; Oh et al., 2018; Zhang D. et al., 2018; Wang Q. et al., 2019). The aromatic heterocyclic rings containing one or two imine groups have a relatively weak electron-withdrawing character, which can be strengthened by introducing additional cyano groups (Tang et al., 2015; Pan et al., 2016; Sasabe et al., 2017; Chen et al., 2018). Although a series of red fluorophores based on pyrazine-2,3-dicarbonitrile has been reported (Gao et al., 2006), the pyrazine-based acceptor hasn't been used to construct a TADF molecule so far. In this paper, the T_1_ energy levels and reduction potentials of various cyano-substituted pyrazines are theoretical investigated. A blue TADF emitter with small Δ*E*_ST_ and relatively large *k*_F_ values is successfully designed and synthesized by employing 2-cyanopyrazine as the acceptor.

## Results and Discussion

**Figure 1:**
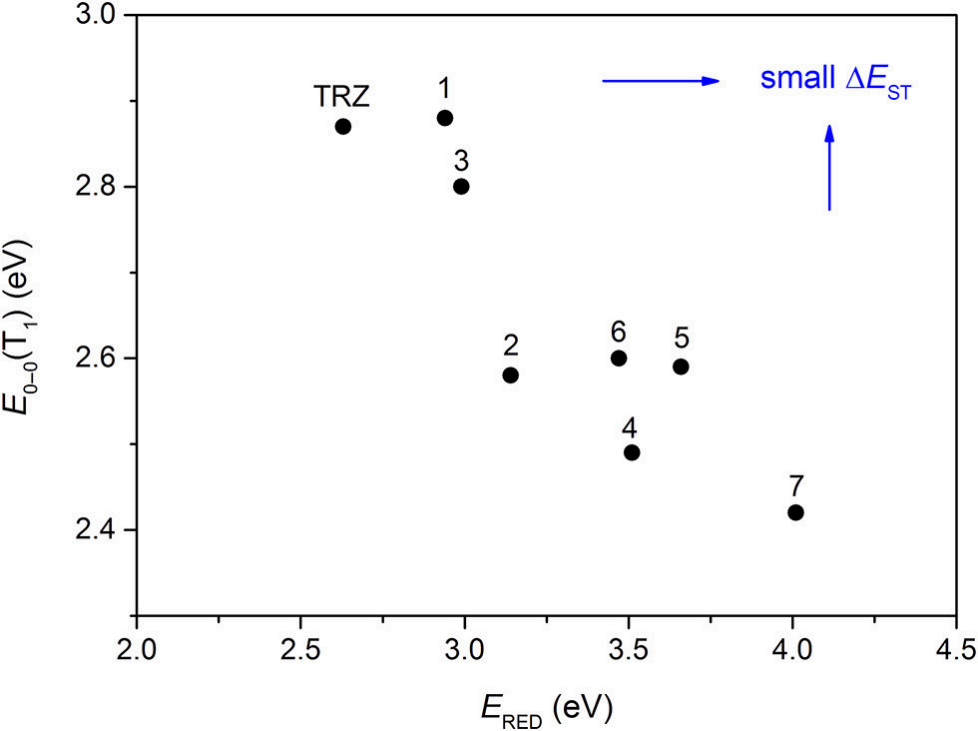
Correlation of calculated *E*_0−0_(T_1_) and *E*_RED_. The structures of the molecules are shown in Table 1.

The CT transition energy is significantly related to the electron-donating ability of the donor and the electron-withdrawing ability of the acceptor in a D-A molecule. To avoid a low-lying locally excited triplet state (^3^LE) existing under the S_1_ (^1^CT), both donor and acceptor moieties should have a limited conjugation length, and the conjugation between donor and acceptor should be broken (Zhang et al., 2014b). The electron-withdrawing capability of pyrazine is weaker than that of 1,3,5-triazine, which is an ideal acceptor for blue and green TADF materials. To enhance the electron-withdrawing capability of pyrazine, one to three cyano groups are attached onto the pyrazine ring in 2-phenylpyrazine, in which the phenyl ring is used as a π-bridge between the donor and acceptor moieties. The calculated zero–zero energy of T_1_ [*E*_0−0_(T_1_)] and the reduction potentials (*E*_RED_) of the substituted pyrazine fragments are listed in Table 1 and compared with those of TRZ (Mazur and Hipps, 1995; Huang et al., 2013; Wang D. et al., 2019). As shown in Figure 1, there is a roughly proportional relationship between *E*_0−0_(T_1_) and *E*_RED_, i.e., reducing the conjugation length of a moiety generally decreases its electron-withdrawing capability. The theoretical *E*_0−0_(T_1_) of 3-phenylpyrazine-2-carbonitrile (Liu Y. et al., 2018) (2.88 eV) is as high as that of TRZ (2.87 eV), while the electron-withdrawing capability of **1** (*E*_RED_ = 2.94 eV) is even higher than that of TRZ (*E*_RED_ = 2.63 eV) (Mazur and Hipps, 1995), indicating that 2-cyanopyrazine is a promising acceptor for blue and green TADF molecules.

**Table 1:** Computed vertical absorption energies (*E*_VA_), zero–zero energies (*E*_0−0_), lowest unoccupied molecular orbital energies (*E*_LUMO_), and reduction potentials (*E*_RED_) of cyano-substituted triazine, pyrimidine, and pyridine moieties.

**No**.	**1**	**2**	**3**	**4**	**5**	**6**	**7**	**TRZ**
Molecular structure^a^	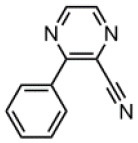	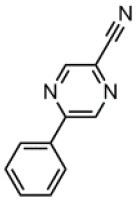	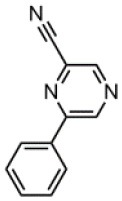	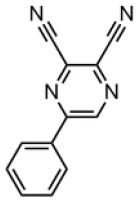	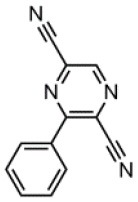	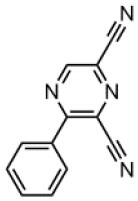	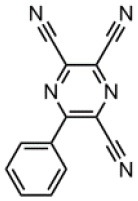	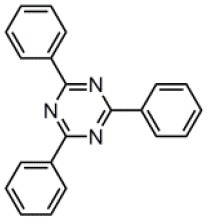
*E*_VA_(T_1_) (eV)^b^	3.03	2.72	2.95	2.63	2.74	2.78	2.57	3.02
*E*_0−0_(T_1_) (eV)^c^	2.88	2.58	2.80	2.49	2.59	2.60	2.42	2.87
*E*_LUMO_ (eV)^d^	−2.45	−2.71	−2.52	−3.18	−3.37	−3.13	−3.81	−2.05
*E*_RED_ (V)^e^	2.94	3.14	2.99	3.51	3.66	3.47	4.01	2.63

a *The geometries are optimized via DFT at the B3LYP/6-311G(d,p) level in vacuum*.

b *Calculated by TD-DFT/B3LYP/6-31G(d) in vacuum*.

c *Calculated from E_VA_(T_1_) with a correlation of E_0−0_(T_1_) = E_VA_(T_1_)/1.02 – 0.09 (Huang et al., 2013)*.

d *Derived from DFT/PBE0/6-311++G(d,p) in acetonitrile*.

e *Referred to an electron in the vacuum state (Mazur and Hipps, 1995) and calculated from the E_LUMO_ with a correlation of E_RED_ = 0.79 × (–E_LUMO_) + 1.01 (Wang D. et al., 2019)*.

**Figure 2:**
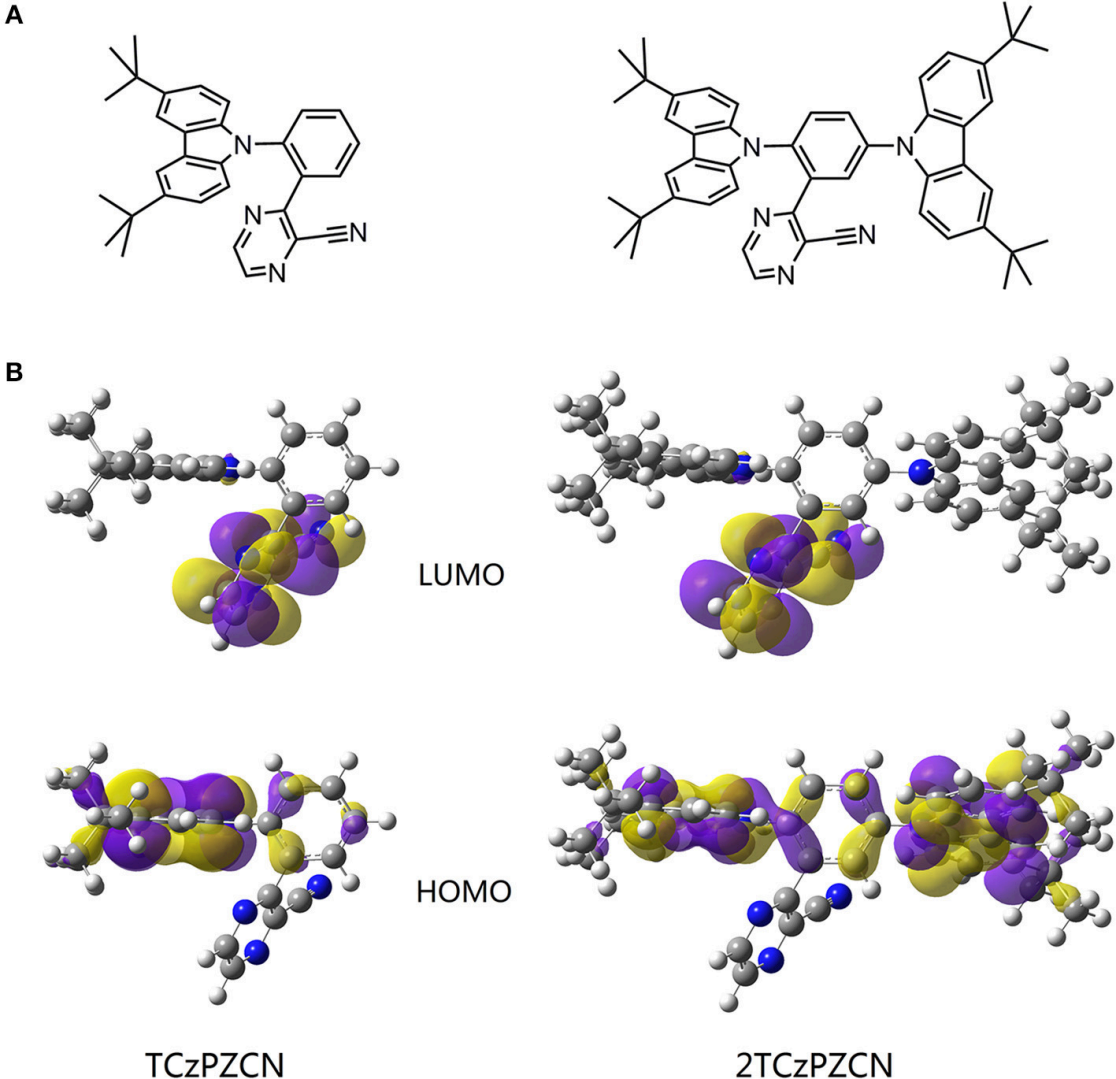
**(A)** Structures of the investigated molecules. **(B)** The highest occupied and lowest unoccupied molecular orbitals (HOMO and LUMO) of the investigated molecules in their S0 state in vacuum. The geometries are optimized via density functional theory (DFT) at the B3LYP/6-311G (d,p) level.

Using 3-phenylpyrazine-2-carbonitrile (Liu Y. et al., 2018) as the π-bridge attached acceptor and 3,6-di-tertbutylcarbazole as the donor, two molecules TCzPZCN and 2TCzPZCN are designed (Figure 2A). TCzPZCN has only one 3,6-di-tertbutylcarbazole donor group, which links to the acceptor group 2-cyanopyrazine via the ortho position of the phenylene (Ph) bridge (Wang R. et al., 2018). The ground-state geometry of TCzPZCN is optimized by DFT/B3LYP/6-31G^*^. The dihedral angle between carbazole donor and Ph-bridge is 69°, while that between 2-cyanopyrazine acceptor and Ph-bridge is 55° (Figure 2B). Such moderate dihedral angles allow a small overlap of the orbitals involved in the CT transition on the Ph-bridge but effectively break the conjugation between the donor and the acceptor. For 2TCzPZCN, there are two 3,6-di-tertbutylcarbazole groups attached to the ortho and meta positions of the Ph-bridge. Although the meta-linked carbazole and the Ph-bridge have a relatively small dihedral angle of 52°, the meta linkage prevents the orbitals on the donor from extending to the acceptor (Figure 2B). Using the *K*-OHF method, a semiempirical descriptor selection method based on time-dependent DFT (Wang et al., 2018), the vertical absorption energies (*E*_VA_) of TCzPZCN and 2TCzPZCN are calculated to be 3.15 and 3.16 eV, respectively. Assuming that their absorption is a 0–1 transition, the commonest transition for TADF emitters in weak polar medium, the *E*_0−0_(S_1_) values of TCzPZCN and 2TCzPZCN in toluene are evaluated to be 2.91 and 2.92 eV, respectively (Huang et al., 2013). The Δ*E*_ST_ and the oscillator strength (*f*) are calculated to be 0.05 and 0.0157 for TCzPZCN, respectively, and 0.05 and 0.0184 for 2TCzPZCN, respectively. The ratios of *f* to Δ*E*_ST_ are among the highest values for the TADF emitters (Δ*E*_ST_ < 0.15 eV) calculated using the *K*-OHF method (Table S1). Using a rough relationship between the theoretical frontier orbital energies and the measured redox potentials (Wang D. et al., 2019), the oxidation potentials (*E*_OX_) of TCzPZCN and 2TCzPZCN are calculated to be 5.46 and 5.41 V, respectively, in dichloromethane, while the *E*_RED_ of TCzPZCN and 2TCzPZCN are calculated to be 2.90 and 2.98 V, respectively, in acetonitrile (Table 2).

**Table 2:** A comparison of theoretical predictions and experimental data on photophysical and electrochemistry of the investigated molecules.

	**Theoretical data**		**Experimental data**	
	**TCzPZCN**	**2TCzPZCN**	**TCzPZCN**	**2TCzPZCN**
**In Toluene**				
*E*_VA_(S_1_) (eV)	3.15	3.16		
*E*_0−0_(S_1_) (eV)^a^	2.91	2.92	2.91	2.82
Δ*E*_ST_ (eV)	0.05	0.05	0.07	0.06
*f*^b^	0.0157	0.0184		
λ_em_ (nm)^c^			490	503
Φ^d^			0.10	0.05
τ_1_/τ_2_/τ_3_ (ns)			0.14/2.6/8.4	0.12/3.2/9.8
**In mCP Film (10 wt%)**				
λ_em_ (nm)			483	493
Φ/Φ_F_			0.47/0.36	0.44/0.16
τ_F_ (ns)			9.7	7.2
τ_TADF_ (μs)			1.9	8.1
*k*_F_ (× 10^7^ s^−1^)			3.7	2.2
**In Dichloromethane**				
*E*_OX_ (V)^e^	5.46	5.41	5.57	5.53
**In Acetonitrile**				
*E*_RED_ (V)^e^	2.90	2.98	2.92	2.95

a *Calculated from E_VA_(S_1_) with a correlation of E_0-0_(S_1_) = E_VA_(S_1_) – 0.24 eV (Huang et al., 2013)*.

b *Oscillator strength*.

c *Emission maximum*.

d *Photoluminescence quantum yield*.

e *Referred to the vacuum state*.

*The theoretical potentials are calculated in the same way as those in Table 1*.

**Figure 3:**
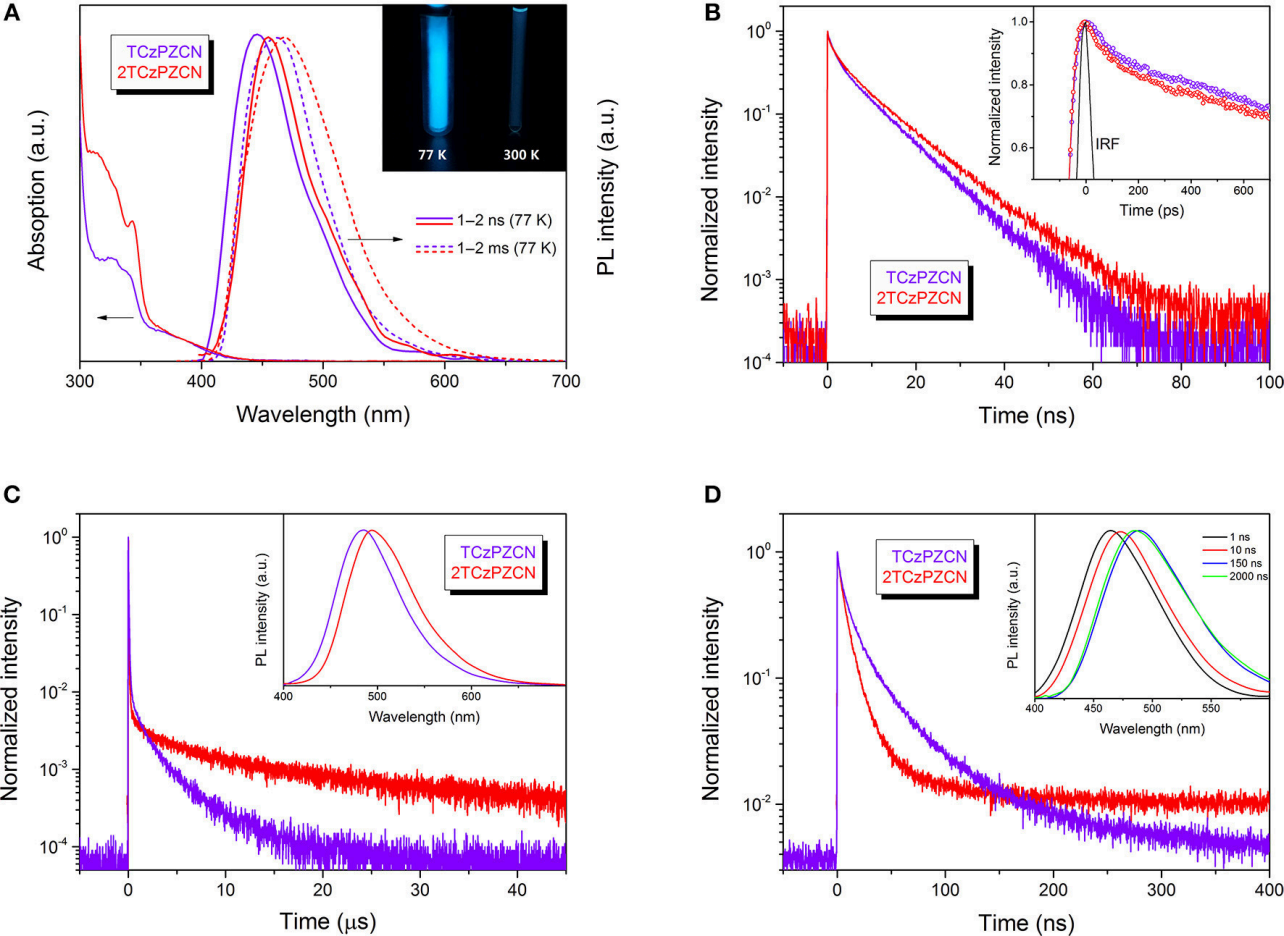
Absorption, emission and transient decay spectra. **(A)** Absorption spectra of the investigated molecules in toluene at room temperature (RT), and fluorescence (1–2 ns) and phosphorescence (1–2 ms) spectra in toluene at 77 K. Inset: A comparison of TCzPZCN in toluene at 77 K and RT under 365-nm ultraviolet irradiation. **(B)** Transient decay spectra of the investigated molecules in toluene at RT. Inset: Instrument response function (IRF). **(C)** Transient decay spectra of the investigated molecules doped into mCP films (10 wt%) measured on a microsecond time scale at RT. Inset: Steady-state emission spectra of the doped films at RT. **(D)** Transient decay spectra of the investigated molecules doped into mCP films (10 wt%) measured on a nanosecond time scale at RT. Inset: Time-resolved emission spectroscopy of the TCzPZCN doped films at RT.

The synthesis of TCzPZCN and 2TCzPZCN is described in the Supplementary Material. Their absorption and emission spectra in toluene and 10 wt% *m*-bis(N-carbazolyl)benzene (mCP) films are presented in Figure 3 and Figure S1. As shown in Figure 3A, the absorption shoulders in the wavelength region of 350–430 nm can be ascribed to the intramolecular charge-transfer (CT) transitions. The fluorescence (1–2 ns component) and phosphorescence (1–2 ms component) spectra in toluene at 77 K are all smooth and broad. The Δ*E*_ST_ values can be estimated from the energy difference between the fluorescence and phosphorescence peaks. The measured Δ*E*_ST_ of 0.07 eV for TCzPZCN and 0.06 eV for 2TCzPZCN are close to the theoretical values (Table 2). From the onset of the fluorescence bands at room temperature (RT; Figure S1), the 0–0 energies of TCzPZCN and 2TCzPZCN in toluene are estimated to be 2.91 and 2.82 eV, respectively, which are also in good agreement with the above theoretical estimation. These two compounds emit brightly at 77 K but dimly at RT with photoluminescence quantum yields (PLQY) < 0.10 (Figure 3A inset and Table 2). In comparison to the emission spectra in solvent glass, those in the fluid solution (Figure S1) exhibit a significant redshift, indicating a correlation between the serious non-radiative decay in RT toluene and the excited-state geometrical relaxation process.

The transient decay spectra in degassed toluene at RT are presented in Figure 3B. No obvious TADF component is observed in the microsecond time range. Besides the single-exponential nanosecond fluorescence decay, a quick decay in the picosecond time scale is recorded by using an ultrafast time-correlated single photon counting (TCSPC). This fast decay could be resolved into two exponentially decaying components with the lifetimes (τ) of 0.14 and 2.6 ns for TCzPZCN and 0.12 and 3.2 ns for 2TCzPZCN. It is reasonable to expect that the non-radiative decay rate (*k*_nr_) is not a constant during the fluorescence decay process. It is known that the excited-state solvation and relaxation process can be completed in a few picoseconds in fluid solution (Castner et al., 1987; Kinoshita and Nishi, 1988), resulting in a very fast non-radiative decay via a so-called free rotor and loose bolt effects (Turro et al., 2010). If the radiative and non-radiative decay rates are constants in the total luminescence process, the *k*_F_ value can be obtained by the following formula:

(1) $$\begin{array}{r} k_{\text{F}}=\Phi_{\text{F}}/\tau_{\text{F}} \end{array}$$

where Φ_F_ and τ_F_ are the PLQY and lifetime of the fluorescence component, respectively. Given that both the *k*_F_ and *k*_nr_ in the first few nanoseconds are the same as the values after that, the PLQYs of these two compounds in toluene will be significantly higher than the observed ones. Consequently, if we calculate *k*_F_ from the measured Φ_F_ and the dominant nanosecond τ with Equation 1, the *k*_F_ value will be considerably underestimated.

In 10-wt%-doped mCP films, TCzPZCN and 2TCzPZCN exhibit blueshifted emission peaks at 483 and 493 nm, respectively (Figure 3C), with respect to that in toluene. Meanwhile, the PLQYs of TCzPZCN and 2TCzPZCN in the doped films increase to 0.47 and 0.44, respectively, owing to the suppression of the collision-induced non-radiative decay (Turro et al., 2010; Zhang et al., 2014a). However, it was previously demonstrated that there is enough free volume in amorphous organic semiconductor films (Sun et al., 2017). The large-amplitude excited-state distortion cannot be fully inhibited in the films, leading to the moderate PLQYs for these films. TADF decay can be observed from the doped films, with a short lifetime of 1.9 μs for TCzPZCN and 8.1 μs for 2TCzPZCN (Figure 3C).

It is known that the solvation effect increases the separation of the electron and hole in a CT state (Sun et al., 2017; Wang and Zhang, 2019) and consequently decreases the fluorescence rate. According to the time-resolved emission spectroscopy shown in Figure 3D, the solvation process in a doped mCP film can last for dozens of nanoseconds, which is much slower than that in fluid solutions (Deng et al., 2019). The fluorescence rate of a TADF emitter in organic thin films decreases gradually in this time region and therefore can have a higher average value than that in solution. Since the fluorescence decays in organic thin films are always best fit by multiple exponentials (Figure 3D), an average lifetime determined from the time the fluorescence intensity decays to 1/e of the initial value (Table 2) is used to calculate the *k*_F_ values. The *k*_F_ value of TCzPZCN in doped mCP films is then calculated to be 3.7 × 10^7^ s^−1^, which is considerably higher than those of the TADF emitters having a Δ*E*_ST_ smaller than 0.1 eV (Table S2). In comparison to TCzPZCN, 2TCzPZCN has a lower *k*_F_ of 2.2 × 10^7^ s^−1^, probably because of the reduced distance between the charge centroids of the donor and acceptor orbitals (Figure 2). According to first-principles calculation, the square root of the CT transition rate is approximately proportional to the effective D/A separation distance and the orbital overlap integral (Phifer and McMillin, 1986; Zhang et al., 2014a).

The oxidation and reduction behaviors of TCzPZCN and 2TCzPZCN are measured by cyclic voltammetry in dichloromethane and acetonitrile, respectively (Figure S2). From the onsets of the quasi-reversible redox couples, the vacuum-state-referenced *E*_OX_ and *E*_RED_ of TCzPZCN are determined to be 5.57 and 2.92 V, respectively, while those of 2TCzPZCN are determined to be 5.53 and 2.95 V, respectively. These potential values are all close to their theoretical ones. In comparison to TRZ-based compounds, TCzPZCN and 2TCzPZCN have higher *E*_RED_ values in favor of electron injection into the emitting layers of their devices.

**Figure 4:**
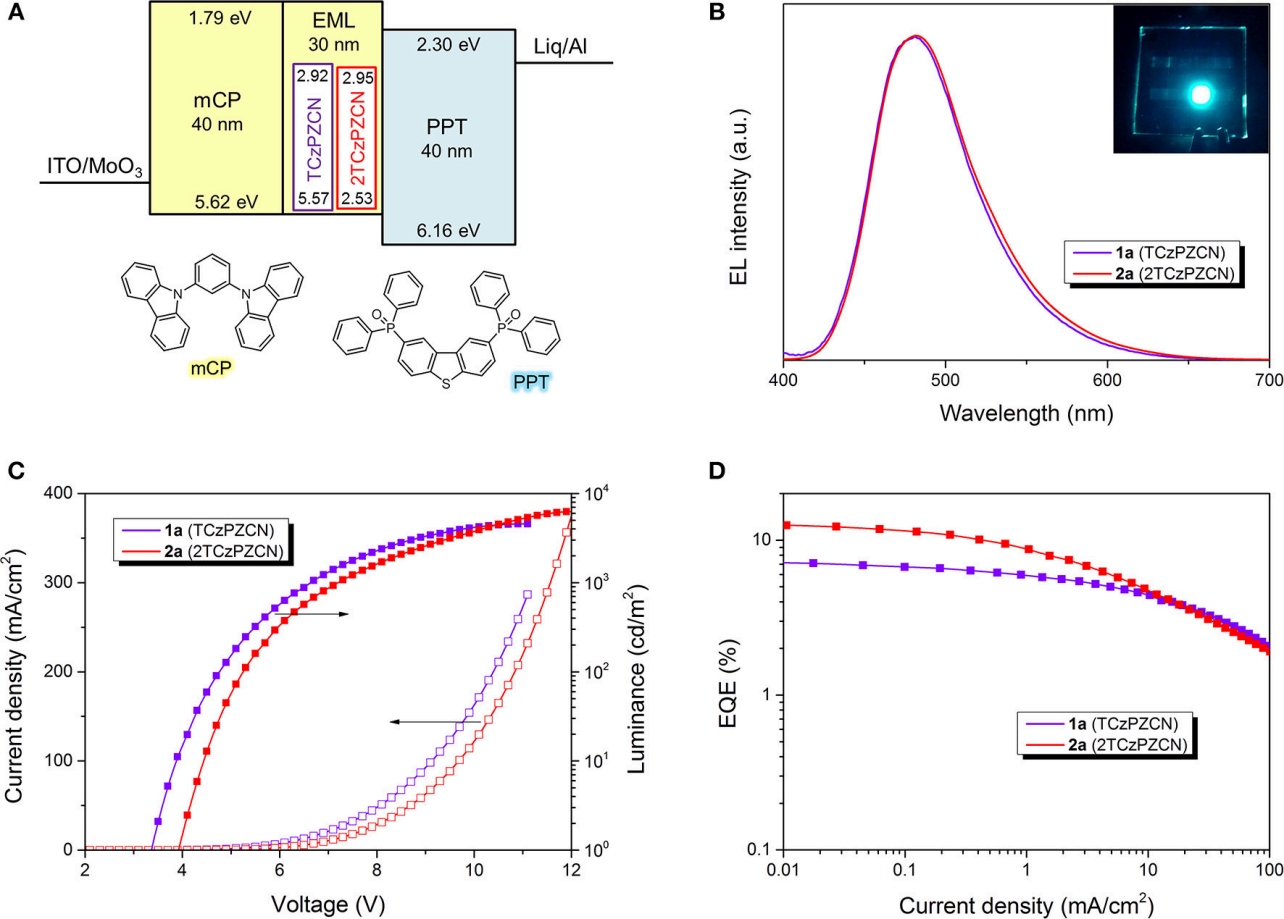
Structures and performance of the thermally activated delayed fluorescence (TADF) organic light-emitting diodes (OLEDs). **(A)** Energy diagram of OLEDs. HOMO and LUMO levels of all compounds were measured by cyclic voltammetry. **(B)** Electroluminescence spectra at 1 mA/cm^2^. Inset: The image of Device **1a**. **(C)** Luminance–current density–voltage characteristics of Devices **1a** and **2a**. **(D)** EQE–current density characteristics of Devices **1a** and **2a**.

Six OLEDs containing TCzPZCN and 2TCzPZCN are fabricated using a very simple device structure, as shown in Figure 4A. The 30-nm-thick emissive layers of the six devices are TCzPZCN or 2TCzPZCN doped mCP films and their neat films (see Table 3). At a doping concentration of 10 wt%, both TCzPZCN- (**1a**) and 2TCzPZCN-based (**2a**) OLEDs display a sky-blue emission with a maximum at 480 nm (Figure 4B). Devices 1a and 2a turn-on at 3.4 and 3.9 V, respectively, and reach a maximum luminance of 4579 and 6257 cd/m2 at 11 and 12 V, respectively (Figure 4C). The maximum external quantum efficiencies (EQEs) of Devices **1a** and **2a** are found to be 7.1 and 12.2%, respectively (Figure 4D), which are both higher than the upper limit of the traditional fluorescent OLEDs (5%). Although the PLQYs of these two compounds are approximate in 10-wt%-doped mCP films, the maximum EQEs of their devices are quite different. The internal quantum efficiency (IQE) of a TADF OLED can approach the PLQY of the emitter only when the internal conversion from S_1_ to S_0_ is the principal deactivation pathway for the emitter (Zhang et al., 2014a). The maximum IQE for Device **1a** is lower than the PLQY of the emissive layer (0.47) when a light out-coupling efficiency of 0.2–0.3 is assumed, indicating that the non-radiative decay at T_1_ for TCzPZCN cannot be ignored in the doped films. In comparison to Device **2a**, Device **1a** shows a reduced EQE roll-off that can be attributed to the short TADF lifetime of 1.9 μs for TCzPZCN in doped film (Zhang et al., 2014a,b).

**Table 3:** Emissive layer (EML) component, turn-on voltage (*V*_on_), maximum luminance (*L*_max_), external quantum efficiency maximum (EQE_max_), emission maximum (λ_max_), full width at half maxima (FWHM), and CIE coordinates of the TADF OLEDs.

**Device**	**EML**	***V*_**on**_ (V)**	***L*_**max**_ (cd/m^**2**^)**	**EQE_**max**_ (%)**	**λ_**max**_ (nm)**	**FWHM (nm)**	**CIE**
**1a**	10 wt% TCzPZCN in mCP	3.4	4,579	7.1	480	70	(0.15, 0.26)
**1b**	30 wt% TCzPZCN in mCP	3.2	5,339	7.6	483	73	(0.15, 0.29)
**1c**	neat TCzPZCN	3.3	6,053	5.4	485	80	(0.17, 0.32)
**2a**	10 wt% 2TCzPZCN in mCP	3.9	6,257	12.2	480	70	(0.15, 0.26)
**2b**	30 wt% 2TCzPZCN in mCP	3.9	7,885	10.4	489	76	(0.17, 0.35)
**2c**	neat 2TCzPZCN	3.7	6,375	4.9	494	80	(0.20, 0.42)

The influence of doping concentration on device performance is shown in Figures S3, S4 and Table 3. The performance of TCzPZCN-based OLEDs is rather insensitive to the doping concentration of the emissive layer owing to the highly twisted configuration of the emitter (Zhang et al., 2015; Cha et al., 2016; Chen X.-L. et al., 2017). The electroluminescence (EL) spectra and EQE–current density curves of 10-and 30-wt%-doped devices almost coincide with each other respectively (Figure S3). Even the undoped device exhibits similar EQE–current density characteristics in the current density range from 1 to 100 mA/cm^2^ with a slightly broader EL spectrum in comparison to the 10-wt%-doped device. In contrast, increasing the doping concentration of 2TCzPZCN-based OLEDs produces clear redshifts of the EL spectrum and decreases the EQE maximum. The lower steric hindrance surrounding the meta-linked carbazole may be responsible for the relatively strong intermolecular interaction between emitters in 2TCzPZCN doped films.

## Conclusion

On the basis of a novel acceptor 2-cyanopyrazine, a type of blue emissive TADF molecule with small Δ*E*_ST_ (< 0.1 eV) is successfully designed and synthesized. The fluorescence kinetics investigation indicates that the non-radiative decay rates of these molecules in toluene are far from constants. The ultrafast fluorescence decay (1/τ) in the first hundred picoseconds after the excitation is related to a significant excited-state structural distortion. In doped mCP films, an *o*-phenylene-bridged 2-cyanopyrazine/3,6-di-tertbutylcarbazole compound (TCzPZCN) shows a fast TADF decay with a lifetime of 1.9 μs, as well as a high fluorescence rate of 3.7 × 10^7^ s^−1^ that can be comparable to those of the TADF emitters having relatively large orbital overlap and Δ*E*_ST_ (>0.2 eV). Although the pyrazine-based TADF emitters in solid films exhibit only moderate quantum yields on PL and EL suffered by the structural distortion process, it is one step toward a TADF emitter with both small Δ*E*_ST_ and large *k*_F_. Additionally, we have demonstrated that 2-cyanopyrazine is a promising acceptor for the construction of blue TADF emitters. By suppressing the structural distortion induced non-radiative decay, efficient pyrazine-based TADF emitters with short TADF lifetime can be expected.

## Data Availability

The datasets generated for this study are available on request to the corresponding author.

## Author Contributions

All authors listed have made a substantial, direct and intellectual contribution to the work, and approved it for publication.

### Conflict of Interest Statement

The authors declare that the research was conducted in the absence of any commercial or financial relationships that could be construed as a potential conflict of interest.
