# Supplementary material for: Pyrazine-Based Blue Thermally Activated Delayed Fluorescence Materials: Combine Small Singlet–Triplet Splitting With Large Fluorescence Rate
Source: Front Chem. 2019 May 21;7:312. doi: 10.3389/fchem.2019.00312 (PMC6536661; doi:10.3389/fchem.2019.00312)
Supplement: Supplementary file 1 [file Table_1.pdf]

Supporting Information:

Pyrazine-Based Blue Thermally Activated Delayed Fluorescence Materials: Combine Small Singlet-Triplet Slipping with Large Fluorescence Rate

Junyuan Liu,[#] Keren Zhou,[#] Dan Wang, Chao Deng, Ke Duan, Qi Ai, Qisheng Zhang**

Table and Figures

Table S1. ΔE_{ST} and oscillator strengths (f) calculated by K-OHF method.[1]

Compounds	ΔE_{ST}	f	$f/\Delta E_{ST}$
CBP	0.59	0.7759	1.315
NPD	0.57	0.3428	0.601
PIC-TRZ	0.4	0.0357	0.089
CC2TA	0.46	0.1879	0.408
PXZ-TRZ	0.02	0.0051	0.255
DPAC-TRZ	0.13	0.0003	0.002
Cz-TRZ	0.45	0.4616	1.026
Cz-TRZ2	0.10	0	0
3Cz-TRZ	0.45	0.3798	0.844
BCz-TRZ	0.42	0.5000	1.190
2CzPN	0.39	0.1302	0.334
4CzPN	0.27	0.0124	0.046
4CzIPN	0.16	0.0971	0.607
4CzTPN	0.16	0.1187	0.742
4CzTPNMe	0.15	0.0057	0.038
DPA-DPS	0.55	0.7691	1.398
DTPA-DPS	0.54	0.848	1.570
DTC-DPS	0.37	0.5206	1.407
DMOC-DPS	0.37	0.4619	1.248
PXZ-DPS	0.05	0.0313	0.626 [a]
DMAC-DPS	0.02	0	0
PPZ-DPS	0.01	0.0009	0.090
DPA-AQ	0.52	0.4878	0.938

DBPA-AQ	0.49	0.6203	1.266
DTC-AQ	0.32	0.3401	1.063
DMAC-AQ	0.02	0.0001	0.005
DPA-Ph-AQ	0.36	0.6255	1.738
DBPA-Ph-AQ	0.35	0.7081	2.023
DTC-Ph-AQ	0.31	0.3579	1.155
DMAC-Ph-AQ	0.03	0.0002	0.007
TCzPZCN	0.05	0.0157	0.314
2TCzPZCN	0.05	0.0184	0.336

[a] For symmetrical D-A-D-type molecules, the oscillator strength in S_1 is about half of that in S_0 due to symmetry breaking (ref 2).

Table S2. Photophysical data of reported TADF materials in doped films.

Compounds	Doped films	Φ_F	τ_F (ns)	k_F ($\times 10^7$ s^{-1})	ΔE_{ST} (eV)	Ref
PIC-TRZ	6 wt% in mCP	0.10	10	1.0	0.11	3
Spiro-CN	6 wt% in mCP	0.05	24	0.2	0.06	4
CC2TA	6 wt% in DPEPO	0.16	27	0.6	0.06	5
PPZ-DPO	10 wt% in mCP	0.12	7	1.7	0.08	6
PPZ-3TPT		0.06	3.5	1.7	0.30	
PPZ-4TPT		0.04	2.8	1.4	0.43	
PPZ-DPS		0.08	10	0.8	0.08	
PXZ-DPS		0.24	14	1.7	0.08	
DMAC-DPS		0.23	21	1.1	0.08	
DPA-AQ	1 wt% in CBP	0.13	4.9	2.7	0.29	2
BBPA-AQ		0.17	5.6	3.0	0.27	
DTC-AQ		0.16	10.4	0.15	0.17	
DMAC-AQ		0.05	16.5	0.30	0.08	
DPA-Ph-AQ		0.54	10.2	5.3	0.24	
BBPA-Ph-AQ		0.54	10.2	5.3	0.22	
DTC-Ph-AQ		0.12	33	0.36		
DMAC- Ph-AQ		0.14	41	0.34	0.07	
DPAC-TRZ	20 wt% in DPEPO	0.36	23.0	1.6	0.16	7
DCz-TRZ		0.78	13.6	5.7	0.17	
*t*Bu3Cz-TRZ		0.54	14.8	3.6	0.15	
BCz-TRZ		0.73	12.3	5.9	0.17	
TCz-TRZ		0.78	12.1	4.7	0.15	
4CzIPN	6 wt% in CBP	0.21	16.5	1.3		8
2PXZ-OXD	6 wt% in DPEPO	0.77	11.2	6.8	0.15	9
2PXZ-TAZ	6 wt% in DPEPO	0.32	23.2	1.4	0.23	
2CzPN	6 wt% in mCP	0.51	16	3.2	0.09	10
DHPZ-2BI	6 wt% in mCBP	0.34	4	8.5	0.19	11

DHPZ-2BN	6 wt% in mCBP	0.17	7	2.4	0.10	
AcPmBPX	6 wt% in mCBP	0.26	20	1.3	0.05	12
PxPmBPX	6 wt% in mCBP	0.21	30	0.7	0.02	
DMAC-TRZ	8 wt% mCPCN	0.55	20.3	2.7	0.05	13
PXZ-TRZ	8 wt% mCPCN	0.47	20	2.4	0.02	
SPXZPO	10 wt% in DPEPO	0.06	8	0.75	0.26	14
DPXZPO		0.1	13	0.77	0.19	
TPXZPO		0.16	20	0.80	0.11	
Ac-OPO	6 wt% in DPEPO	0.22	18	1.2	0.03	15
Ac-OSO		0.30	29	1.0	0.06	
Py2	8 wt% in mCPCN	0.68	10.4	6.5	0.19	16
Py5		0.70	10.7	6.6	0.18	
Py56		0.53	26.7	2.0	0.03	
Pm2		0.56	15.7	3.6	0.09	
Pm5		0.56	23.7	2.4	0.04	
Cz-TRZ1	6 wt% in DPEPO	-	-	10.3	0.43	17
Cz-TRZ2		-	-	2.7	0.07	
Cz-TRZ3		-	-	7.7	0.17	
Cz-TRZ4		-	-	5.1	0.15	
BP-phIDID	6 wt% in PMMA	-	6.6	8.2	0.20	18
Tria-phIDID		-	7.3	8.1	0.12	
fppyBTPA	8 wt% in mCPCN	0.38	48	0.79	~0	19
fppyBCzP		0.15	11.4	1.3	0.24	
dfppyBTPA		0.50	46	1.1	~0	
ClPPM	1.5 wt% in mCBP	0.60	21	2.9	0.06	20
BrPPM		0.59	20	3.0	0.07	
PXZPM	6 wt% in mCBP	0.75	21	3.6	0.08	
DCzBN1	10 wt% in DPEPO	0.18	4	4.5	0.31	21
DCzBN2		0.28	6.6	4.2	0.22	
DCzBN3		0.27	6.8	4.0	0.26	
DCzBN4		0.39	12.8	3.0	0.14	
PXZ-PCN	10 wt% in CBP	0.44	12.9	3.4	0.01	22
Bis-PXZ-PCN		0.26	17.7	1.5	0.04	
Tri-PXZ-PCN		0.26	16.2	1.6	0.05	
CzDCNPy	20 wt% in DPEPO	0.44	13.3	3.3	0.32	23
tBuCzDCNPy		0.47	17.2	2.7	0.27	
DTRZ-DI	25 wt% in TCTA: Bepp2	0.32	20	1.6	0.03	24
TRZ-DI		0.37	21	1.8	0.02	
BuCz*o*B	10 wt% in DPEPO	0.15	47.1	0.32	0.08	25
BuCzMe*o*B	20 wt% in DPEPO	0.16	42.6	0.38	0.09	
Cz*o*B		0.23	41.2	0.56	0.12	
CzMe*o*B		0.14	27.1	0.52	0.13	
CzOMe*o*B		0.12	22.9	0.52	0.14	

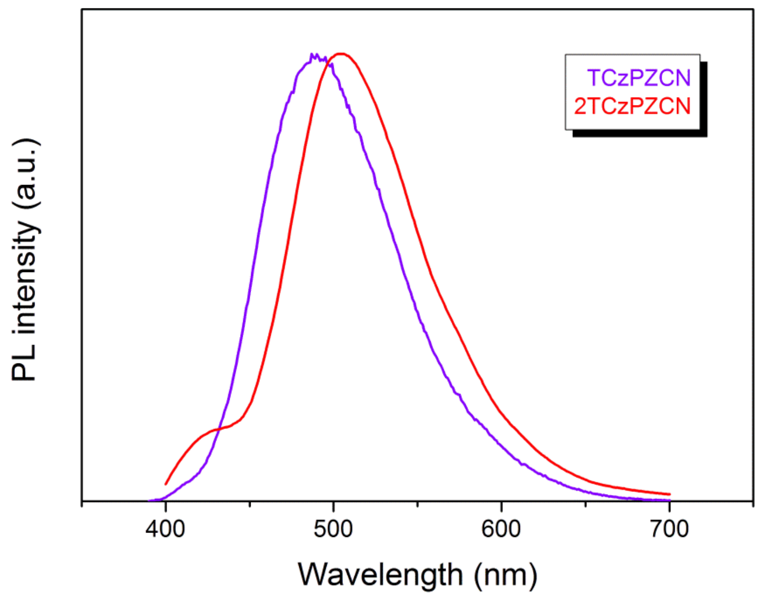

Figure S1. Emission spectra of the investigated molecules in toluene at RT.

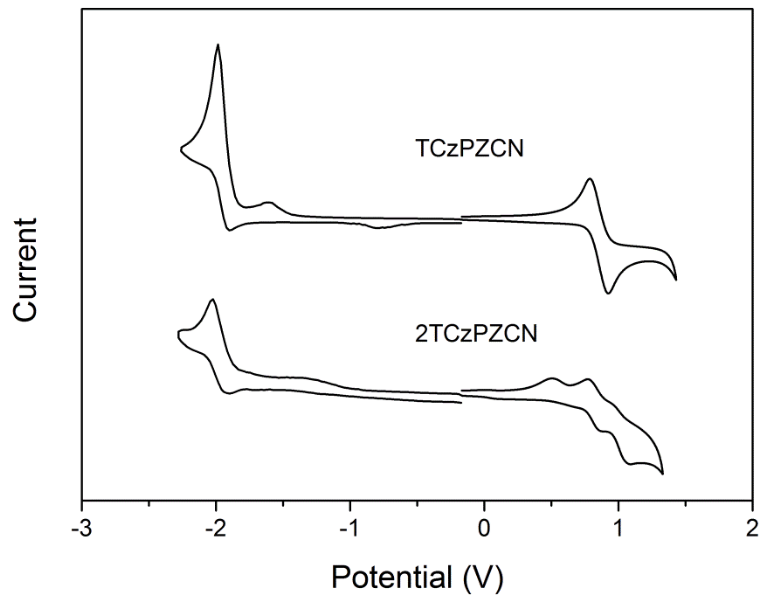

Figure S2. Cyclic voltammogram for the oxidation (in dichloromethane) and reduction (in acetonitrile) of the investigated molecules at RT. Scan rate 100 mV s^{-1} in 0.1 M TBAP.

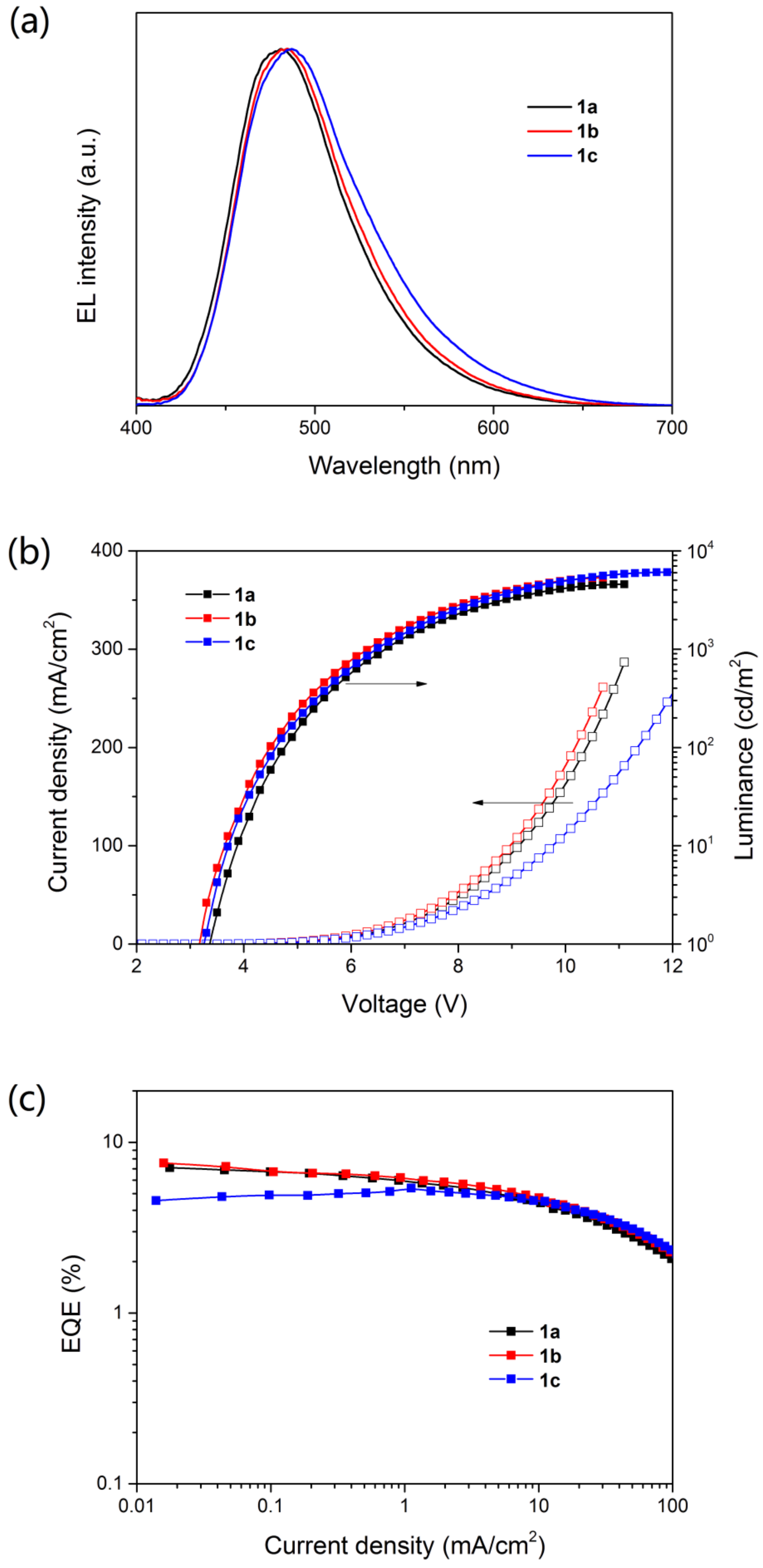

Figure S3. Electroluminescence spectra (**a**, at 1 mA/cm^2), luminance–current density–voltage characteristics (**b**), and EQE–current density characteristics (**c**) of Device **1a**, **1b** and **1c**.

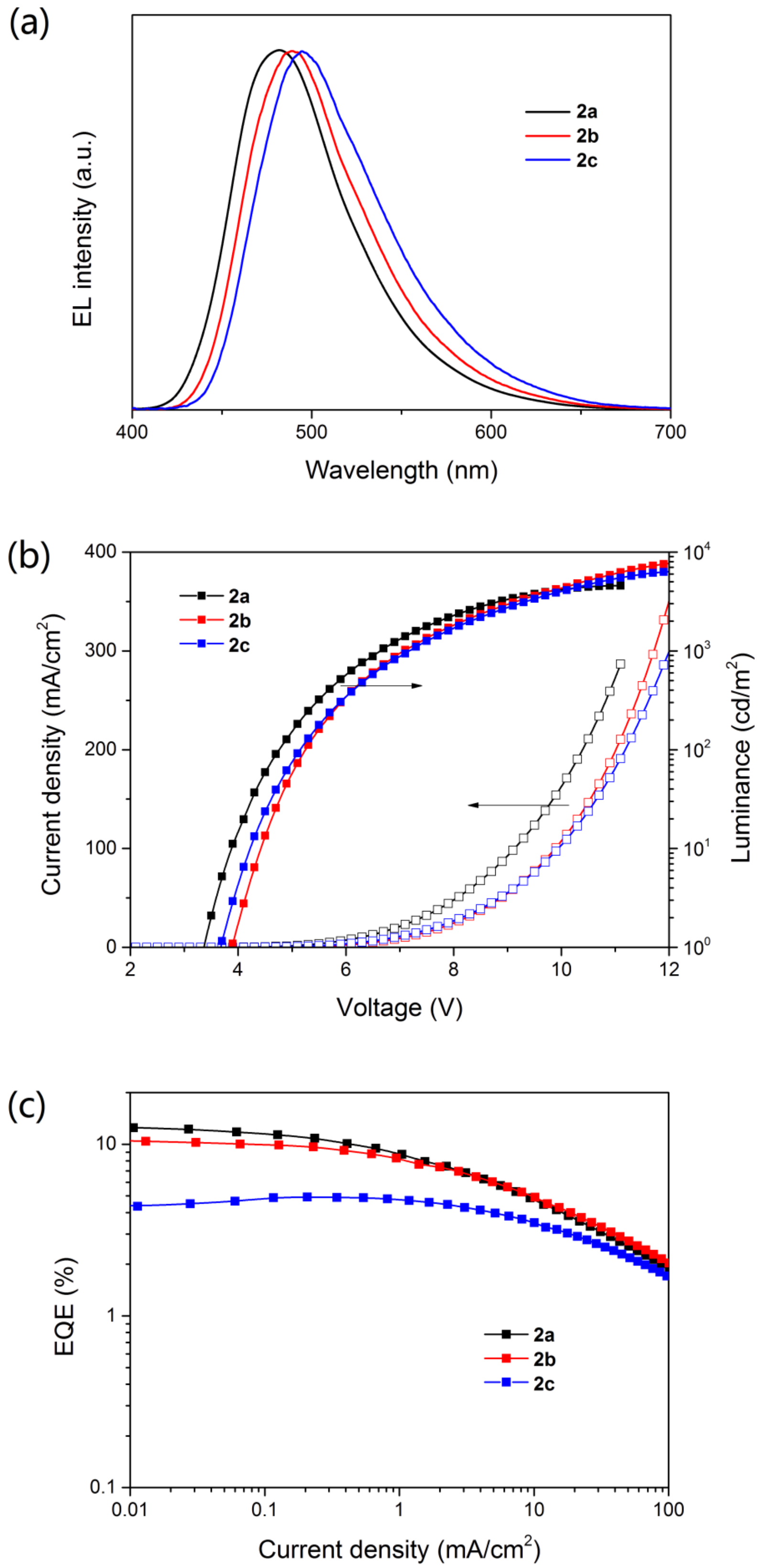

Figure S4. Electroluminescence spectra (**a**, at 1 mA/cm^2), luminance–current density–voltage characteristics (**b**), and EQE–current density characteristics (**c**) of Device **2a**, **2b** and **2c**.

Experimental

1. Materials and synthesis:

General: All solvents and starting materials were purchased from commercial resources and were used as received unless otherwise stated. The intermediates 3,6-di-tert-butyl-9H-carbazole, trimethyl borate and 3-chloropyrazine-2-carbonitrile were purchased from Bidepharm Co., Ltd. and were used without further purification. The OLED materials MoO_3, mCP, DPEPO, PPT and Liq were purchased from Jilin Optical and Electronic Materials Co., Ltd. and were used without further purification. Nuclear magnetic resonance spectroscopy (NMR) were recorded on a Bruker Avance III 400 spectrometer (^1H: 400 MHz and ^{13}C: 100 MHz) at room temperature using $CDCl_3$ as solvent and tetramethylsilane (TMS) as internal reference. Mass spectra measurements were performed on a IT-TOF (Shimadzu, Japan) equipped with an ESI source in positive ion mode. Accurate mass determination was corrected by calibration using the sodium trifluoroacetate clusters as reference. Elemental analyses (C, H, N) were carried out with a Vario MICRO cube (Elementar).

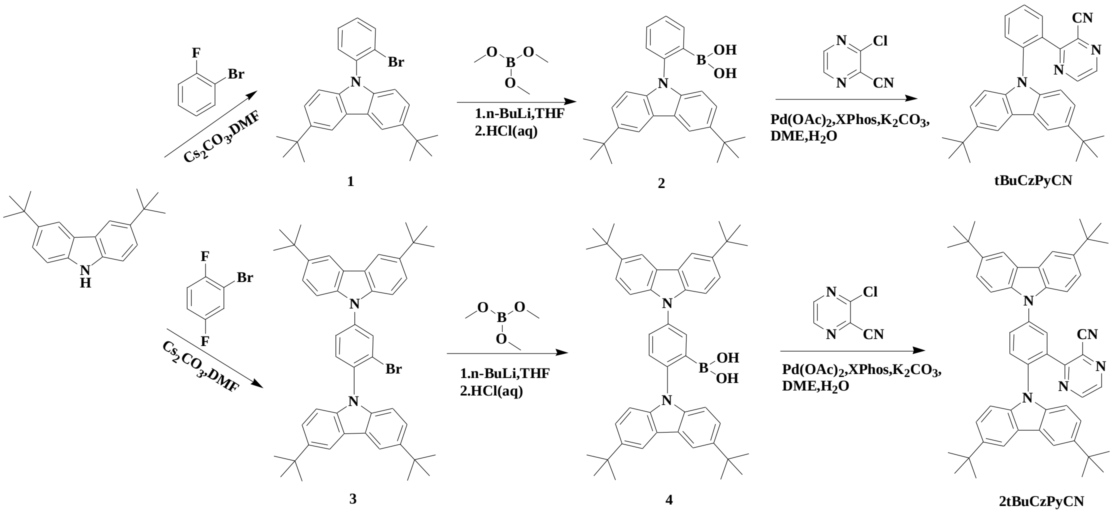

2-(3,6-di-tert-butyl-carbazole)-bromobenzene **1**

To an anhydrous DMF solution (15 mL) of 3,6-Di-tert-butylcarbazole (2.79 g, 10 mmol), Cs_2CO_3 (6.52 g, 20 mmol) was added at room temperature. After the mixture was degassed with nitrogen for 10 minutes, 1-bromo-2-fluorobenzene (1.31 ml, 12 mmol) was added, and then the mixture was heated to reflux for 24 hours. The reaction was quenched by adding H_2O. The reaction mixture was cooled to room

temperature and extracted with dichloromethane. After the solvent was evaporated under a reduced pressure, the residue was purified by column chromatography (DCM/PE=1/10) to afford a white solid (4.01 g, 9.2 mmol) in a 92% yield. ^{1}H NMR (400 MHz, $CDCl_3$)：δ=8.14 (s, 2H), 7.84 (d, *J*=8 Hz, 1H), 7.51-7.31 (m, 5H), 6.99 (d, *J*=8 Hz, 2H), 1.44 (s, 18H).

2-(3,6-di-tert-butyl-carbazole)-phenylboronic acid **2**

A solution of *n*-butyllithium (7.27 mL of 2.2 M in *n*-hexane, 8.0 mmol) was added to a stirring solution of **1** (3.47 g, 8 mmol) in dry THF (100 mL) at -78 ℃ for a period of 15 min and stirred at -78℃ for additional 1 hour under a nitrogen atmosphere. Triisopropylborate (2.32 mL, 24.0 mmol) was added and the mixture was allowed to warm up to room temperature. After 5 hours stirring, the reaction was quenched by adding 1M HCl in ice bath. The reaction mixture was extracted with ethyl acetate. The organic layer was washed with water, followed by brine solution and dried over $MgSO_4$. This organic layer was filtered off and concentrated to give the crude product, which was purified by column chromatography (DCM/PE=1/1) to give **2** (3.12 g, 78%) as a white solid. ^{1}H NMR (400 MHz, $CDCl_3$)：δ=8.17-8.10 (m, 3H), 7.62-7.52 (m, 2H), 7.43 (d, *J*=8 Hz, 2H), 7.21 (d, *J*=8 Hz, 2H), 4.54 (s, 2H), 1.44 (s, 18H).

TCzPZCN

A saturated aqueous K_2CO_3 solution (10 mL) was added to a solution of 2-chloro-3-methylpyrazine (1.12 g, 8.0 mmol) and **2** (2.78 g, 7.0 mmol) in ethylene glycol diethyl ether (20 mL). The mixture was stirred under a nitrogen flow for 15 min. $Pd(OAc)_2$ (0.079 g, 0.35 mmol) and 2-(dicyclohexylphosphino)-2',4',6'-tri-i-propyl-1,1'-biphenyl (0.29 g, 0.7 mmol) were added, and the mixture was refluxed for 48 h. The reaction mixture was cooled and extracted with ethyl acetate. The organic layer was washed with water, followed by brine solution and dried over $MgSO_4$. This organic layer was filtered off and concentrated to give the crude product, which was purified by column chromatography (DCM/PE=1/3) to give **TCzPZCN**

(1.84 g, 58%) as a yellow solid. ^{1}H NMR (400 MHz, $CDCl_3$): δ=8.33 (d, *J*= 2 Hz, 1H), 8.27 (d, *J*= 2 Hz, 1H), 7.99 (d, *J*= 4 Hz, 2H), 7.82 (dd, *J*=4, 8 Hz, 1H), 7.74(td, *J*=4, 8 Hz, 1H), 7.67-7.62 (m, 2H), 7.34(dd, *J*=2, 8 Hz, 1H), 7.09 (d, *J*=8 Hz, 2H), 1.40 (s, 18H). ^{13}C NMR (100 MHz, $CDCl_3$): δ=156.52, 145.79, 143.05, 139.48, 137.18, 133.61, 131.33, 129.76, 129.51, 128.34, 123.60, 123.40, 116.02, 115.49, 109.50, 34.68, 31.96. HRMS (FAB+): *m/z* calcd. for $C_{31}H_{30}N_4$ 458.2470, found 458.2455. Anal Calcd for $C_{31}H_{30}N_4$: C, 89.19%; H, 6.59%; N, 12.22%. Found: C, 89.17%; H, 6.57%; N, 12.25%.

2,5-di-(3,6-di-tert-butyl-carbazole)-bromobenzene **3**

A procedure similar to that used for **1** produced white solid in 91% yield. ^{1}H NMR (400 MHz, $CDCl_3$): δ=8.18-8.17 (m, 4H), 8.11 (d, *J*=4 Hz, 1H), 7.75-7.72 (m, 1H), 7.63 (d, *J*=12 Hz, 1H), 7.54 (m, 4H), 7.52 (d, *J*=4 Hz, 1H), 7.50 (d, *J*=4 Hz, 1H), 7.21 (d, *J*=8 Hz, 1H), 1.49 (s, 18H), 1.43 (s, 18H).

2,5-di-(3,6-di-tert-butyl-carbazole)-phenylboronic acid **4**

A procedure similar to that used for **2** produced white solid in 85% yield. ^{1}H NMR (400 MHz, $CDCl_3$): δ=8.35 (d, *J*=2 Hz, 1H), 8.17 (d, *J*=2 Hz，4H), 7.78 (dd, *J*=2, 4 Hz, 1H), 7.52-7.48 (m, 6H), 7.42 (d, *J*=8 Hz, 1H), 7.21 (d, *J*=8 Hz, 2H), 1.49 (t, *J*=4 Hz, 36H).

2TCzPZCN

A procedure similar to that used for **TCzPZCN** produced yellow solid in 45% yield. ^{1}H NMR (400 MHz, $CDCl_3$): δ=8.33-8.30 (m, 2H), 8.18-8.17 (m, 2H), 8.02-7.96 (m, 4H), 7.84 (d, *J*=8 Hz, 1H), 7.67 (d, *J*=8 Hz, 2H), 7.57-7.55 (m, 2H), 7.43-7.40 (m, 2H), 7.24 (d, *J*=8 Hz, 2H), 1.49 (s, 18H), 1.43 (s, 18H). ^{13}C NMR (100 MHz, $CDCl_3$): δ=156.05, 145.85, 143.70, 143.33, 139.59, 138.83, 138.28, 138.21, 135.16, 131.03, 129.03, 124.06, 123.78, 123.53, 121.31, 116.43, 116.14, 109.48, 109.28, 34.82, 34.72, 32.01, 31.96. HRMS (FAB+): *m/z* calcd. for C51H53N5 735.4301, found 735.4374. Anal Calcd for C51H53N5: C, 82.23%; H, 7.26%; N,

9.52%. Found: C, 82.19%; H, 7.28%; N, 9.51%.

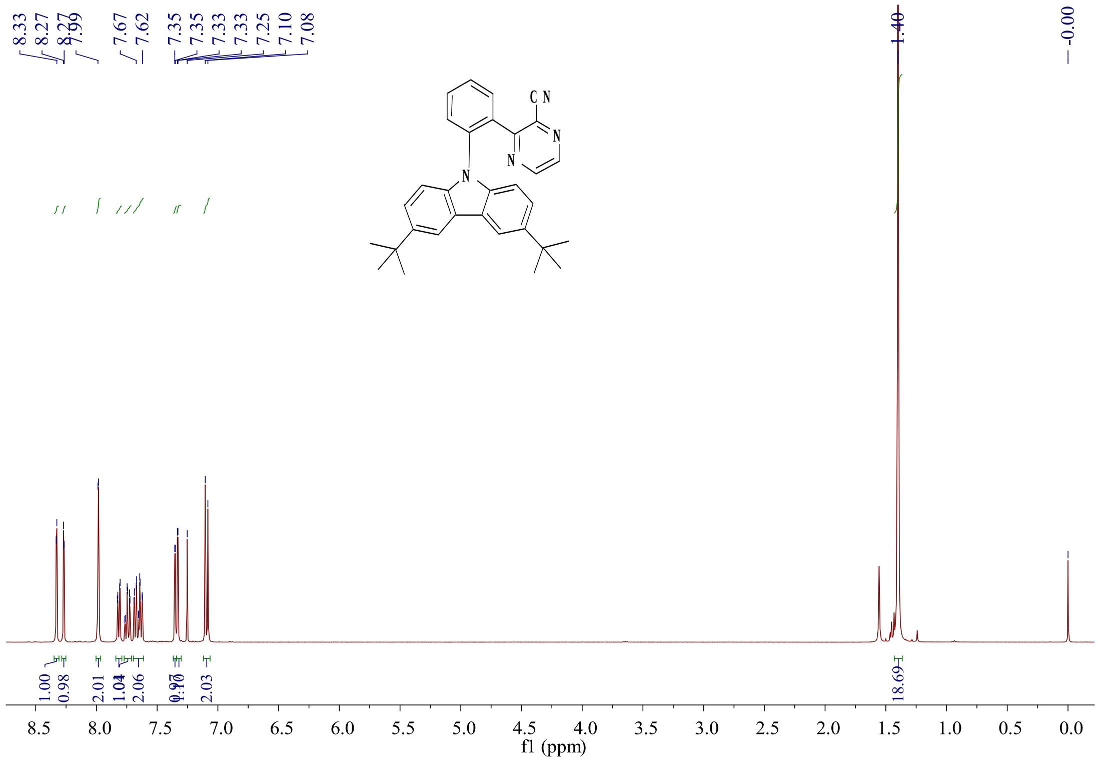

Figure S5. ^{1}H NMR spectrum of **TCzPZCN**.

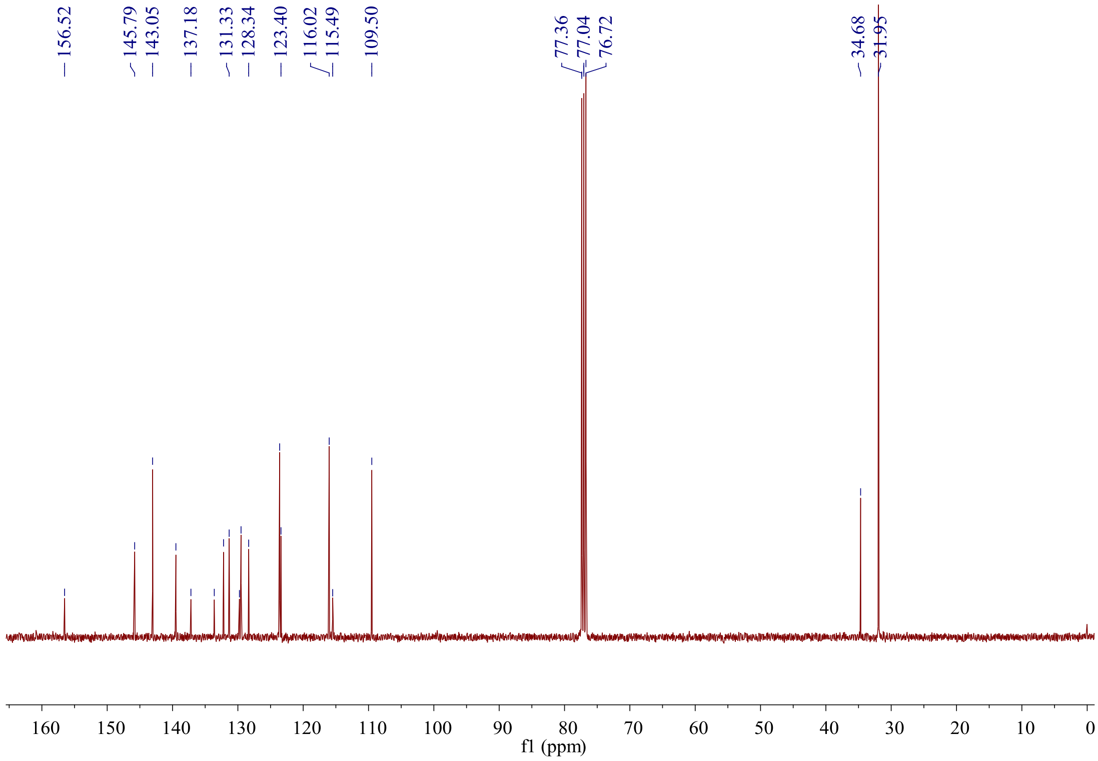

Figure S6. ^{13}C NMR spectrum of **TCzPZCN**.

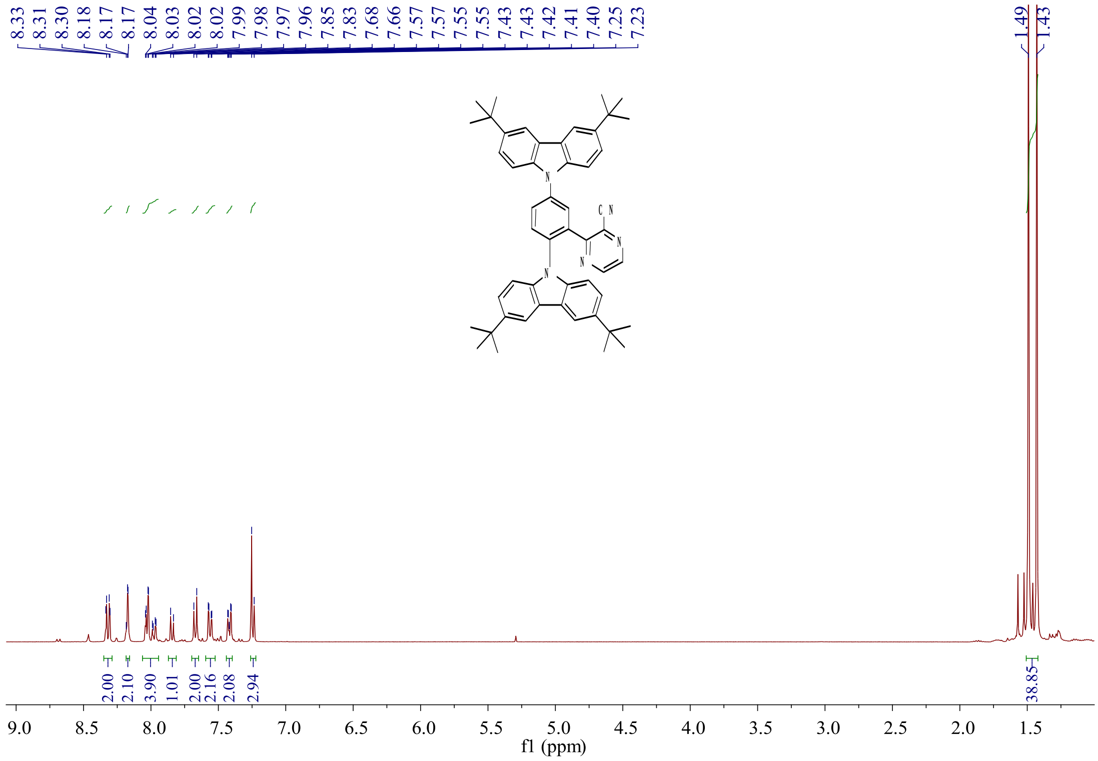

Figure S7. ^{1}H NMR spectrum of **2TCzPZCN**.

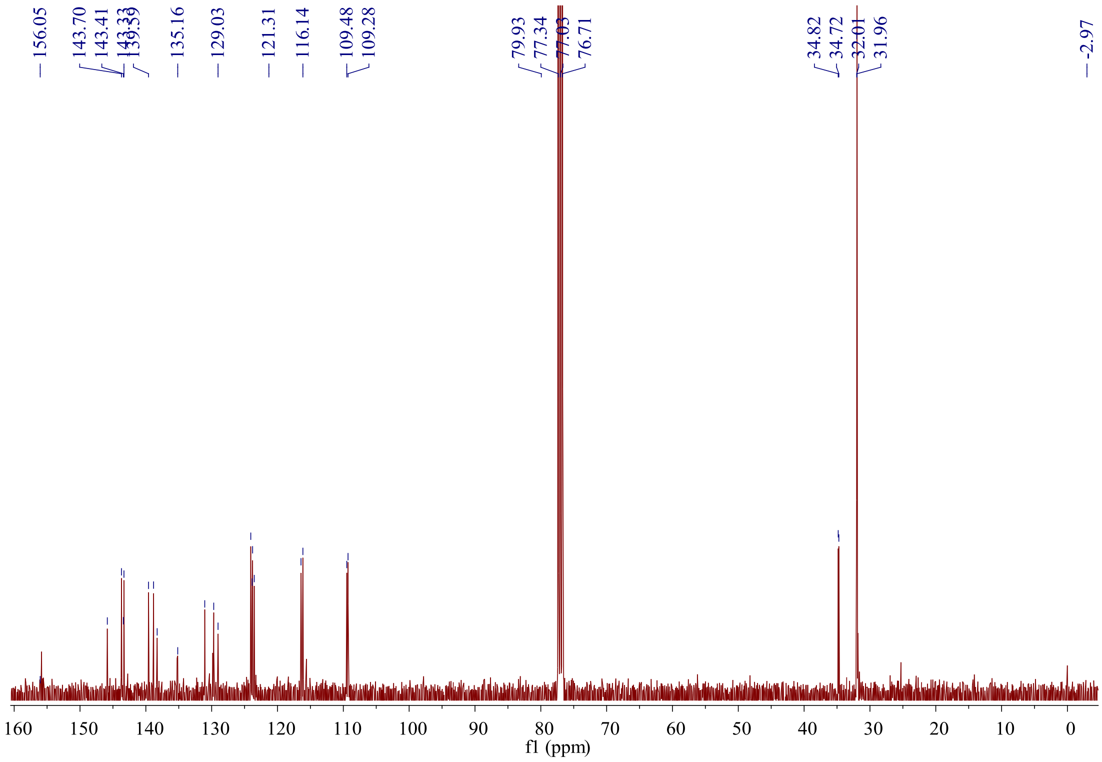

Figure S8. ^{13}C NMR spectrum of **2TCzPZCN**.

2. Photoluminescence measurements:

The absorption and emission spectra were recorded using a Shimadzu UV-2600 spectrophotometer and a PTI QM-40 spectrofluorometer, respectively. The photoluminescence quantum yields were obtained by the QM-40 equipped with an integrating sphere attachment. The low-temperature fluorescence (1-2 ns) and phosphorescence (1-2 ms) spectra were collected by the strobe technique and an electric shutter, respectively, using the PTI spectrofluorometer equipped with a PTI nitrogen laser (GL-3300, λ = 266 nm, pulse width$\sim$1 ns, pulse energy = 1.45 mJ) and a liquid nitrogen dewar. The transient decay spectra were measured by the TCSPC technique, using a Horiba DeltaFlex modular lifetime measurement system equipped with a diode laser (λ = 371 nm, pulse width $\approx$ 50 ps, repetition rate = 20.00 kHz). The fluorescence decay curves were analyzed using the deconvolution software.

3. Electrochemical measurements:

Cyclic voltammetry were performed using a CHI 600E electrochemical analyzer in a gas-tight three-electrode cell at room temperature. A glassy carbon working electrode (Φ = 5.0 mm), a platinum wire auxiliary electrode, and an Ag/Ag^+ reference electrodes (0.1 M $AgNO_3$, 0.1 M tetrabutylammonium hexafluorophosphate in acetonitrile) were used. The oxidation and reduction processes were measured by scanning the potential at a scan rate of 100 mV/s in dichloromethane and acetonitrile, respectively, with 0.1 M tetrabutylammonium hexafluorophosphate (TBAP) as supporting electrolyte. The solutions were degassed by purging with N_2 gas for approximately 3 min before the measurement.

4. Quantum Chemical Calculations:

All calculations were performed using the Gaussian 09 program package.[26] The geometries in the ground state were optimized via DFT calculations at the B3LYP/6-311G(d,p) level in vacuum. Frequency analysis was used to confirm that the structures are at the local minima of the potential surfaces. The vertical excitation energies of the moieties were calculated at the TD-DFT/B3LYP/6-31G(d) in vacuum,

while the excitation energies and the oscillator strength of the TADF molecules were calculated at the TDA-PBEα_o/6-31+G(d) level in vacuum using the *K*-OHF method.[1] The optimal fractions of exact exchange (α_o) for **TCzPZCN** and **2TCzPZCN** are 0.37 and 0.39, respectively. The theoretical redox potentials were corrected from the frontier orbital energies which were derived from DFT/PBE0/6-311++G(d,p) in acetonitrile or dichloromethane.[27]

5. Device Fabrication and Measurements:

After the pre-cleaned indium tin oxide (ITO) glass substrates were treated with ozone for 15 min, the inorganic, organic and metal layers were thermally evaporated onto the substrates in an inert chamber under a pressure of 2×10^{-4} Pa. The deposition rates were 0.1 Å/s for MoO_3 layer, 1-2 Å/s for organic layers, and 4 Å/s for Al layer. The current density, voltage and luminance characteristics of the devices were measured in ambient air with a Keithey 2400 Source meter and a Keithey 2000 Source multimeter equipped with a calibrated silicon photodiode. The electroluminescence spectra were recorded using a multichannel spectrometer (PMA12, Hamamatsu Photonics). Assuming Lambertian emission, the external quantum efficiency can be calculated from the luminance, current density, and EL spectrum.

References

[1] C. Wang, C. Deng, D. Wang, Q. Zhang, *J. Phys. Chem. C*, **2018**, *122*, 7816−7823.

[2] Q. Zhang, H. Kuwabara, W. J. Potscavage, S. Huang, Y. Hatae, T. Shibata, C. Adachi, *J. Am. Chem. Soc.*, **2014**, *136*, 18070–18081.

[3] A. Endo, K. Sato, K. Yoshimura, T. Kai, A. Kawada, H. Miyazaki, C. Adachi, *Appl. Phys. Lett.*, **2011**, *98*, 083302.

[4] T. Nakagawa, S.-Y. Ku, K.-T. Wong, C. Adachi, *Chem. Commun.*, **2012**, *48*, 9580–9582.

[5] S. Y. Lee, T. Yasuda, H. Nomura, C. Adachi, *Appl. Phys. Lett.*, **2012**, *101*, 093306.

[6] Q. Zhang, B. Li, S. Huang, H. Nomura, H. Tanaka, C. Adachi, *Nat. Photonics*, **2014**, *8*, 326–332.

[7] Z. Liu, F. Cao, T. Tsuboi, Y. Yue, C. Deng, X. Ni, W. Sun, Q. Zhang, *J. Mater. Chem. C*, **2018**, *6*, 7728.

[8] H. Uoyama, K. Goushi, K. Shizu, H. Nomura, C. Adachi, *Nature*, **2012**, *492*, 234–238.

[9] J. Lee, K. Shizu, H. Tanaka, H. Nomura, T. Yasudaabc, C. Adachi, *J. Mater. Chem. C*, **2013**, *1*,

4599–4604.
[10] K. Masui, H. Nakanotani, C. Adachi, *Org. Electron.*, **2013**, *14*, 2721–2726.
[11] J. Lee, K. Shizu, H. Tanaka, H. Nakanotani, T. Yasuda, H. Kajic, C. Adachi, *J. Mater. Chem. C*, **2015**, *3*, 2175–2181.
[12] S. Y. Lee, T. Yasuda, I. S. Parka, C. Adachi, *Dalton Trans.*, **2015**, *44*, 8356–8359.
[13] W.-L. Tsai, M.-H. Huang, W.-K. Lee, Y.-J. Hsu, K.-C. Pan, Y.-H. Huang, H.-C. Ting, M. Sarma, Y.-Y. Ho, H.-C. Hu, C.-C. Chen, M.-T. Lee, K.-T. Wong, C.-C. Wu, *Chem. Commun.*, **2015**, *51*, 13662–13665.
[14] C. Duan, J. Li, C. Han, D. Ding, H. Yang, Y. Wei, H. Xu, *Chem. Mater.*, **2016**, *28*, 5667–5679.
[15] S. Y. Lee, C. Adachi, T. Yasuda, *Adv. Mater.*, **2016**, *28*, 4626–4631.
[16] K.-C. Pan, S.-W. Li, Y.-Y. Ho, Y.-J. Shiu, W.-L. Tsai, M. Jiao, W.-K. Lee, C.-C. Wu, C.-L. Chung, T. Chatterjee, Y.-S. Li, K.-T. Wong, H.-C. Hu, C.-C. Chen, M.-T. Lee, *Adv. Funct. Mater.*, **2016**, *26*, 7560–7571.
[17] L.-S. Cui, H. Nomura, Y. Geng, J. U. Kim, H. Nakanotani, C. Adachi, *Angew. Chem. Int. E*d., **2017**, *56*, 1571–1575.
[18] C. H. Ryoo, I. Cho, J. Han, J. Yang, J. E. Kwon, S. Kim, H. Jeong, C. Lee, S. Y. Park, *ACS Appl. Mater. Interfaces*, **2017**, *9*, 41413–41420.
[19] Y.-J. Shiu, Y.-T. Chen, W.-K. Lee, C.-C. Wu, T.-C. Lin, S.-H. Liu, P.-T. Chou, C.-W. Lu, I.-C. Cheng, Y.-J. Lien, Y. Chi, *J. Mater. Chem. C*, **2017**, *5*, 1452–1462.
[20] Y. Xiang, Y. Zhao, N. Xu, S. Gong, F. Ni, K. Wu, J. Luo, G. Xie, Z.-H. Lu, C. Yang, *J. Mater. Chem. C*, **2017**, *5*, 12204–12210.
[21] C.-Y. Chan, L.-S. Cui, J. U. Kim, H. Nakanotani, C. Adachi, *Adv. Funct. Mater.*, **2018**, *28*, 1706023.
[22] Z. Chen, Z. Wu, F. Ni, C. Zhong, W. Zeng, D. Wei, K. An, D. Ma, C. Yang, *J. Mater. Chem. C*, **2018**, *6*, 6543–6548.
[23] L. Gan, K. Gao, X. Cai, D. Chen, S.-J. Su, *J. Phys. Chem. Lett.*, **2018**, *9*, 4725–4731.
[24] K. J. Kim, G. H. Kim, R. Lampande, D. H. Ahn, J. B. Im, J. S. Moon, J. K. Lee, J. Y. Lee, J. Y. Lee, J. H. Kwon, *J. Mater. Chem. C*, **2018**, *6*, 1343–1348.
[25] Y. H. Lee, S. Park, J. Oh, S.-J. Woo, A. Kumar, J.-J. Kim, J. Jung, S. Yoo, M. H. Lee, *Adv. Opt. Mater.*, **2018**, *6*, 1800385.
[26] M. J. Frisch, et al. Gaussian 09, D.01; Gaussian, Inc.: Wallingford, CT, **2009**.
[27] D. Wang, S. Huang, C. Wang, Y. Yue, Q. Zhang, *Org. Electron.*, **2019**, *64*, 216–222.
